# Adaptation and Implementation of a Multi-Family Group Psychoeducational Intervention for Parents of Children with Autism: A Pilot Study

**DOI:** 10.3390/jcm14072307

**Published:** 2025-03-28

**Authors:** Ioanna G. Tsiouri, Angeliki Gena

**Affiliations:** 1 School of Philosophy, Department of Educational Studies, National and Kapodistrian University of Athens, 10679 Athens, Greece; agena@otenet.gr; 2 Psychiatric Clinic, University Hospital of Larissa, 41334 Larissa, Greece

**Keywords:** autism spectrum disorder, group psychoeducation, family functioning, family rituals, family burden, stigma management

## Abstract

**Background/Objectives:** A relatively small number of studies have evaluated the effectiveness of interventions designed to ameliorate family burden and to improve family functioning for families with a child with ASD. This study aims to investigate whether a long-term multi-family group psychoeducational intervention, originally developed for families including a member with a psychiatric disorder, can assist the parents of children with ASD to improve family functioning, support family rituals, and ease family burden; to understand the etiology, the characteristics, and treatment options for ASD; and to manage social and self-stigmatization. **Method**: We compared an intervention group (N = 3 couples—6 parents) with a waitlist control group (N = 3 couples—6 parents) by administering psychometric scales to evaluate the effectiveness of the intervention on (a) family functioning, (b) family rituals, and (c) family burden. Qualitative analysis of pre- and post-intervention semi-structured interviews assessed (a) the participants’ understanding of the nature, causes, and treatments for ASD and (b) the management of social and self-stigmatization in families with a child with ASD. **Results:** Quantitative pre- and post-test group comparisons, as well as qualitative thematic analysis, revealed significant decreases in all parameters under study for the treatment group. **Conclusions:** Our findings provide pilot evidence that long-term group psychoeducation, originally designed for families including a member with a psychiatric disorder, may provide an efficacious treatment choice toward improving the general functioning of families with a child with ASD. Systematic replications of this psychoeducational intervention merit attention.

## 1. Introduction

Autism spectrum disorder (ASD) is classified as a neurodevelopmental disorder characterized by the persistent impairment of communication and social interaction; restricted, repetitive patterns of behavior, interests, or activities; and sensory sensitivities [1]. Recent epidemiological studies estimate ASD prevalence worldwide to be around 1%, consistently increasing over the past 15–20 years [2,3]. There are also much higher estimates of its prevalence; for example, 2.85% of 8-year-old students in the USA have been diagnosed with ASD [4]. The active involvement of family members in the implementation of therapeutic protocols has been shown to enhance the development and prognosis of children with ASD [5,6,7]. Nevertheless, it has been extensively documented that parents who raise a child with ASD are chronically exposed to high levels of stress [8], due to various psychosocial challenges stemming from the child’s social, communicative, and behavioral difficulties and from limited access to comprehensive therapeutic interventions and rehabilitation services for individuals with ASD [9,10,11,12,13]. Chronic stress is detrimental to the parents’ health and their quality of life and has been associated with somatic symptoms, anxiety, depression, and poorer perceived general health compared to the general population [12,14]. Chronic stress has adverse effects not only on the parents but also on the family members diagnosed with ASD [6,15]. It is inarguable that people with ASD are not only the recipients of stress but also impose stress on other family members [16]. Expressed emotion (EE) (which refers to negative emotional intensity in the family context and is a risk factor for relapse) is another factor with an adverse impact on the family dynamics and on the person with ASD since it has been proven to exacerbate or maintain behavior problems and autistic symptomatology [17,18,19]. On the other hand, longitudinal studies have shown that parents’ warmth and positive attitude toward their children with ASD is associated with decreasing behavior problems [6,20].

The cumulative effects of emotional strain, financial difficulties, social challenges, and psychosomatic symptoms are referred to as “family burden” [21,22,23,24,25,26]. Researchers distinguish between objective burden, defined as manifested disorder-associated costs to families (e.g., financial issues), and subjective burden, defined as each family’s interpretation of the hardships associated with facing a serious disorder [25]. Family burden or the difficulties associated with raising a child with ASD may impose difficulties on the family’s daily functioning and its overall social adjustment, such as the family’s engagement in social rituals, traditions, ceremonial activities, vacations, and recreational social activities, which are all important for maintaining family cohesion and stability [27].

It has also been demonstrated that raising a child with ASD imposes a strain on the marital relationship—leading to the disruption of family cohesion—and also has a negative impact on the interaction and communication among the family members themselves (including the extended family), and between family members and their neighbors and friends, which may lead to psychosocial disengagement or marginalization of the family [10,28]. Nevertheless, aside from evaluating the burden and strain imposed on families, it is important to explore parameters that may improve the quality of family functioning. The McMaster model of family functioning [29] provides a comprehensive description of six dimensions pertaining to the functioning of the family system and, thus, may be used for families with a member with a chronic disability [30]. The dimensions of the McMaster model are evaluated using the Family Assessment Device [29].

Another adversity that families raising a child with ASD need to cope with is stigma. Described as an attribute that is deeply discrediting, with components such as labeling, stereotyping, separating, status loss, and discrimination, social stigma leads to poorer quality of life for families including children with ASD [31]. Self-stigma is the most debilitating type of stigmatization since the person who experiences self-stigma is adopting an “illness identity”—a devalued view of oneself that overshadows every other identity [32].

Despite the identification of a host of difficulties that may be associated with raising a child with ASD, and the undoubted benefits of parent training and support services, a relatively small number of studies have evaluated the effectiveness of interventions designed to ameliorate the parents’ psychological difficulties, to reduce family burden, and to improve family functioning and family atmosphere for families including a child with ASD [12,33,34,35,36]. Stressful life events may often not be avoidable, yet improving coping mechanisms that help reduce family burden may be a realistic goal for families of individuals with ASD.

Due to the scarcity of studies addressing the improvement of family functioning, and recognizing the benefits of having a warm and supportive family atmosphere, it was considered important to explore the literature for interventions where their effectiveness is well documented in clinical populations other than ASD. The literature provides extensive empirical evidence on the effectiveness of a specific intervention protocol in the field of mental disorders, namely, family psychoeducation, which was developed and introduced by Falloon and Liberman [37] and addresses families including a member with schizophrenia. Family psychoeducation entails didactic and therapeutic elements that aim to inform family members about the illness and to guide them on how to improve family functioning, to handle and cope with the illness, and to manage social and self-stigma. The psychoeducational process typically includes: (a) briefing about the illness, (b) training in problem-solving, (c) practicing effective communication, and (d) learning to assert one’s needs [30,38].

Empirical studies and meta-analyses—conducted with populations from the USA, Australia, and Europe—have demonstrated that parent support programs, implemented with the parents of children with ASD, lead to improved parental psychosocial outcomes [39]. In addition, participation in psychoeducational programs can reduce family burden, improve coping strategies, enhance family organization and coherence, and reduce social and self-stigma [30,40,41,42,43]. Family psychoeducation formats vary, including long vs. brief programs, single-family vs. multi-family group programs, and peer-led vs. professional-led programs [6].

Family psychoeducation has also been shown to reduce family stress and improve outcomes in other populations with various diagnoses, such as cancer, asthma, and mood disorders [44,45,46,47,48,49,50,51,52,53]. Pertaining to the diagnosis of ASD, Dawalt et al. [6] provided a preliminary evaluation of a controlled multi-family group psychoeducation intervention addressing the parents of adolescents with ASD, yielding promising outcomes, since improvements were noted in parental depressive symptoms and problem-solving skills following treatment. Nevertheless, the majority of psychoeducation programs address the children’s needs and aim to support the children with ASD rather than their parents [27,54], who need to increase their competence in parenting a child with ASD, overcome social isolation, and decrease their stress levels [27]. Thus, the need to design psychoeducational interventions that primarily address parental functioning in families with a child with ASD remains prevalent.

Hence, the originality of the present study lies in: (a) the systematic adaptation and application of an evidence-based psychoeducational intervention, designed originally for families including a member with schizophrenia, to parents of children with ASD; and (b) the extensive focus of this psychoeducation program on the improvement of the systemic properties of the family of a child with ASD (parental communication and affective responsiveness skills, problem-solving skills, adherence to family rituals, social and self-stigma management, and stress management). Finally, considering the international focus on parent support programs, the importance of this study lies in the need to assess the efficacy of programs in countries such as Greece, where there is only a handful of studies regarding psychoeducational services for parents and no intervention studies for the parents of children with ASD.

For the purposes of the present study, it was hypothesized that a long-term multi-family group psychoeducational intervention, which was originally designed for the parents of individuals with schizophrenia [30,38], would be an effective intervention for parents of children with ASD. Specifically, the research questions of this study were whether this group psychoeducational intervention would improve: (a) family functioning (problem-solving, communication, roles, behavior control, affective responsiveness, and involvement, per the McMaster family model), assessed by the Family Assessment Device [27]; (b) family engagement in rituals and routines, assessed using the Family Rituals Scale [55]; and (c) family burden (psychological, financial, health, and social hardships), assessed using the Family Burden Scale [25]; (d) understanding of the nature, causes, and treatments for ASD and (e) management of social stigma and self-stigma, based on pre- and post-intervention qualitative data collected by the participants through individualized semi-structured interviews.

## 2. Materials and Methods

### 2.1. Participants

Six couples—the parents of children diagnosed with ASD, who were all attending educational and therapeutic programs at the Institute of Systemic Behavior Analysis (ISBA), located in Athens, Greece, and at the Day Center Hara II (DCH II) in Larissa, Greece—participated in this study. The participation of the parents in the study was voluntary. The demographic characteristics of the participants and their offspring are depicted in Table 1.

### 2.2. Settings and Researchers

The following participant inclusion criteria were implemented: (a) all participants’ parents were Greek, residing in the geographic regions of either Athens or Larissa, and fluent speakers of Greek; (b) all parents had a minimum of a high-school diploma; (c) the parents shared the same household—they were not separated or divorced—and were the primary caregivers of the children with ASD who resided with their families at the time of the intervention; (d) participants did not have a psychiatric history nor were they receiving psychiatric medication at the time of the study. According to the inclusion criteria for children with ASD, they had to: (a) be diagnosed with ASD by an independent professional in the past 4–6 years prior to the study, thus, their mean age was 7.11; (b) receive a minimum treatment duration of 3 h per day, 5 days per week of intensive special education services; (c) were under no medication at the time of the study.

The study had a duration of over 12 months. The members of the treatment group met on a biweekly basis at the ISBA, where psychoeducational group sessions were held in an office area. The control group received individual psychological counseling, which entailed active listening and psychological support techniques based on the systemic family therapy approach, provided at the Day Center Hara II by a psychologist who specializes in autism. Couples from the control group were registered on a waitlist to receive group psychoeducation in the year to come.

The psychoeducational treatment sessions were conducted by the two authors of the present article, who are highly experienced clinical psychologists, both holders of a doctoral degree in Behavior Analysis, and certified in family psychotherapy (systemic and behavioral approaches).

The first author served as the primary observer, being responsible for data collection and the analysis of all research sessions. There were also two psychology undergraduate students who served as secondary independent observers and who were trained systematically for the purposes of the present study.

### 2.3. Assessment Instruments

#### 2.3.1. Standardized Assessment Measures

The effectiveness of treatment was assessed by comparing the performances of the two groups across both quantitative and qualitative measures. Treatment and control group participants were tested pre-and post-intervention with the following self-reported questionnaires, each measuring a separate family parameter: family functioning (Family Assessment Device), family rituals and routines (Family Rituals Scale), and family burden (Family Burden Scale). For the purposes of the qualitative assessment, we conducted individualized semi-structured interviews with each participant pre- and post-treatment on the following topics: (a) understanding of the nature, causes, and treatments of ASD and (b) ways of managing the social stigma and self-stigma associated with raising a child with ASD.

##### Family Functioning

The Family Assessment Device (FAD) was used to assess family functioning [27]. FAD is a paper-and-pencil, 60-item, self-administered questionnaire that assesses one’s perception of his/her family across seven dimensions: (a) Problem-Solving: skills to manage issues that threaten the functional capacity of the family and the integrity of the family unit. (b) Communication: verbal interactions among family members that permeate clear messages (c) Roles: concretely stated and equally distributed assignment of responsibilities among family members, providing nurturance and support to one another, promoting personal development for each family member, and following up on whether the tasks assigned are carried out responsibly. (d) Affective Responsiveness: the extent to which each family member has affective reactions that are congruent with the social context. (e) Affective Involvement: the extent to which family members are interested in and show respect for each other’s actions or concerns. Family well-being corresponds with intermediate levels of involvement; low and high scores for involvement are associated with dysfunction. (f) Behavioral Control: the standards for the behavior that the family sets for its members, which may be flexible, rigid, indifferent, or chaotic. The questionnaire is scored in the direction of dysfunction, meaning that scoring 4 reflects high levels of dysfunction. The Greek version of the FAD shows high subscale internal consistency (Cronbach’s *a* > 0.7) [56]. The FAD subscales are psychometrically sound (Cronbach’s *a* from 0.72 to 0.92). Cutoff scores for normal family functioning are ≥ 2 for General Family Functioning, 2.3 for Family Roles, 2.2 for Communication, Problem-solving, and Affective Responsiveness, 2.1 for Affective Involvement, and 1.9 for Behavior Control.

##### Family Rituals

The Family Rituals Scale (FRS) [55] is a self-administered, 11-item questionnaire that measures three forms of family activities that improve members’ participation in family rituals and routines: (a) family traditions on religious holidays, (b) family celebrations and trips, and (c) family routines. The questionnaire is scored in the direction of dysfunction, with 11 indicating the regular practice of family rituals and 44 showing the absence of family rituals. The cutoff score for FRS is 18. The scale shows adequate internal consistency (Cronbach’s alpha = 0.89).

##### Family Burden

The Family Burden Scale (FBS) [25] is a twenty-three-item self-reported questionnaire that assesses the burden experienced by the caregivers of individuals with psychiatric disorders. It measures burden across four dimensions: (a) effect on everyday activities and general socializing, (b) aggressive or violent incidences, (c) effect on the physical and mental health of the caregiver, (d) effect on financial status/financial problems because of the patient’s psychiatric problem. The first, second, and fourth parameters measure objective burden, whereas the third is a measure of subjective burden. The scale is scored in the direction of dysfunction and presents adequate internal consistency (Cronbach’s *a* = 0.85). The cutoff score is 24.

#### 2.3.2. Qualitative Assessment

##### Semi-Structured Interview

A semi-structured interview was conducted with each one of the participating parents (treatment and control group), both before and after the intervention. The interview was based on a set of six open-ended questions, organized around two main topics: (a) parents’ understanding of the nature of their offspring’s disorder (e.g., the etiology, symptoms, treatment, and prognosis of ASD) and (b) self- and social stigma management (e.g., what they think of themselves as parents of an individual with ASD, or what they believe other people think of them as parents with an offspring with ASD).

### 2.4. Design and Procedure

A controlled group trial, quasi-experimental design with pre- and post-measures was used to assess the effectiveness of the treatment procedure. Couples were assigned to two groups and matched by city of origin (Athens or Larissa), years of education, age, and the number of years passed from the initial diagnosis of their child. The treatment group (N = 6, three couples who received intensive psychoeducational therapy, and the control group (N = 6, three couples)) received standard counseling, provided by a psychologist at a daycare center. Taking into consideration the small sample sizes per group of participants (N = 6) [57], a *t*-test for analyzing independent samples was conducted, showing equality of means for age, years of education, and years of diagnosis between the two groups (*p* > 0.2), thus making the two groups relatively homogeneous.

The study was conducted according to the Declaration of Helsinki, and the research protocol was approved by the research ethics committee of the National and Kapodistrian University of Athens (NKUA) and of the ISBA (project identification code and date: 78/7 September 2017). Parents were contacted, in person, to discuss the purpose and procedures of the study. Written informed consent was obtained from all 12 parents prior to the beginning of the study.

All six couples (treatment and control group) were pre- and post-tested using both quantitative and qualitative means of assessment. Standardized tests were self-administered in a private room in paper and pencil format. Semi-structured interviews yielded the data used for qualitative analysis pertaining to the participants’ (a) understanding of the nature, causes, and treatments of ASD, and (b) ways of managing the social stigma and self-stigma associated with raising a child with ASD. The interview was audiotaped for data-collection purposes and had no time limit (average interview time: 60 min). The offspring received specialized ASD treatment services throughout the study at the ISBA (treatment group) and DCH-II (control group).

The three couples who participated in the treatment group received 23 biweekly 90-minute sessions conducted by two experienced clinical psychologists, who were also the study coordinators; they are both holders of doctoral degrees in Applied Behavior Analysis for children with ASD and are both certified in family psychotherapy (systemic and behavioral approaches).

The structure and the content of the psychoeducation program were based on the behavioral-family-therapy protocol developed by Falloon and his associates [30,58,59]. Proper adjustments of the protocol were made by the study coordinators to address the needs of children with ASD, based on parental reports and relevant studies regarding the parents of children with ASD [9,10,11,12,13,60,61]. Parental reports were systematically drawn from semi-structured individual interviews, during which the parents answered questions posed by the coordinators and addressed difficulties that the families encountered at that time. During all treatment and focus group sessions, parents and therapists sat in a circle to ensure full attendance at the group process by all group members. The content of the intervention after adaptations was as follows: (a) to inform parents about the nature, possible risk factors, and psychoeducational interventions for ASD, (b) to assist them in developing coping skills that would help them deal with social and self-stigma, (c) to train them in techniques for improving communication and problem-solving, and (d) to assist them in developing behavior management skills. To meet the protocol’s aims, several techniques were used, including modeling, role-playing, positive feedback, and promoting generalization through homework assignments. Educational material associated with the content of the therapeutic sessions was also provided, either printed or in reference to internet websites. The content and structure of the program are summarized in Table 2.

### 2.5. Data Collection and Analysis

For data collection and analysis purposes, all sessions were audio-taped, scored, and analyzed by independent observers to ensure adherence to the implementation of the treatment protocol (treatment fidelity) and the reliability of treatment outcomes. Analysis of the quantitative and qualitative data will be presented in this section separately and in detail. Data were also collected on communication and problem-solving skills training of the parents for interobserver agreement purposes.

#### 2.5.1. Quantitative Analysis

SPSS 18.0 J for Windows was used to assess the questionnaire scores. Two types of nonparametric statistical tests were conducted. Specifically, for the purpose of comparing before-and-after treatment effects within each group, mean scores before and after treatment were compared using the Wilcoxon matched-pairs signed-rank test, since this is a test suggested for repeated measurements on single small samples when the population from which they are drawn cannot be assumed to be normally distributed [57]. Mean score comparisons within groups for the total scores were conducted for the Family Assessment Device (FAD), the Family Rituals Scale (FRS), and the Family Burden Scale (FBS). For the FAD and the FBS, post hoc before-and-after within-group comparisons using the Wilcoxon matched-pairs signed-rank test were conducted for all their subscales’ mean scores. To compare the mean scores between groups (the psychoeducation group and the standard-care control group) before and after treatment on the same three questionnaires, the non-parametric statistical-hypothesis Mann–Whitney U test was conducted; this is a non-parametric significance test that is frequently used for equal small-sample sizes.

#### 2.5.2. Qualitative Analysis

Patterns in the answers that were provided during the structured interviews before and after the intervention, as received from both the treatment and the control groups, were identified, coded, and categorized. The identified themes were the following: (a) understanding ASD (etiology, characteristics, treatment, and prognosis) and (b) social and self-stigma management.

## 3. Results

The quantitative and qualitative results of the present study are presented separately. The quantitative outcomes are the product of statistical analysis comparing pre- and post-treatment data collected from the three questionnaires (FAD, FRS, and FBS) using within- and between-group comparisons. Qualitative outcomes are yielded from the systematic identification, coding, and categorization of data collected during semi-structured interviews conducted with each participating parent (pre-and post-treatment) data collected from within- and between-group comparisons that were conducted with each of the six couples of parents before and after the intervention. The IOA for standardized-measure outcomes (quantitative data) and for the qualitative data derived from semi-structured interviews and focus groups was 100%. For treatment fidelity purposes, interobserver agreement (IOA) data were collected in 70% of the treatment sessions. The IOA ranged from 80 to 100%, with an average of 92%.

### 3.1. Quantitative Outcomes

#### 3.1.1. Family Assessment Device

Table 3 shows the mean scores (SD) on the subscales of the FAD before and after intervention within the treatment group (N = 6) and within the control group (N = 6), as well as the cutoff scores on each subscale for normal family functioning. The results of the Wilcoxon matched-pairs signed-ranks test analysis (*z*-values and *p*-values) are presented for each subscale mean score difference within each of the two groups. The pre-intervention mean scores indicated family functioning at pathological levels across all family dimensions for both groups, with the exception of *Emotional Involvement,* which was within the normal range (cutoff = 2.10) for both treatment (M = 1.92; SD = 0.2) and control group (M = 2.01; SD = 0.3). *Problem-Solving* was referred to as the most abnormal dimension of family functioning for both the treatment group (M = 2.83; SD = 0.37) and control group (M = 2.65; SD = 0.2; cutoff = 2.20).

For the treatment group, the Wilcoxon matched-pairs signed-rank test comparisons of all pre- and post-treatment mean subscale scores showed a statistically significant (*p* < 0.05) decrease across all seven family dimensions, except for Emotional Involvement (*p* = 0.6) (which was already within normal family functioning levels (M < 2.10) prior to intervention). Additionally, the post-intervention mean scores for the treatment group dropped under the cutoff scores across all family functioning dimensions, indicating normal levels of family functioning following the intervention, with one exception. The mean family subscale scores for Behavior Control remained at marginally dysfunctional levels (M =1.98; SD = 0.1; cutoff score = 1.90). For the control group, there were no statistically significant mean differences (*p* < 0.05) on the Wilcoxon matched-pairs signed-rank test comparisons on any of the pre- and post-treatment mean subscale scores. The mean scores of the control group remained at dysfunctional levels (higher than the cutoff scores) across all family functioning dimensions, except for the Emotional Involvement subscale (M = 2.06; SD = 0.4), which was already within the normal range before the intervention.

Table 3 also depicts the results of the Mann–Whitney U-test (average ranks and *p*-values) comparing the mean scores of all family-functioning subscales at pre- and post-test between the treatment and control groups. Statistical significance was set at *p* < 0.05. There were no statistically significant differences between the two groups at pre-test (*p* > 0.05), showing no between-group systematic differences in any of the family-functioning dimensions before intervention. When the two groups were compared post-treatment, however, the treatment group’s average rank scores in all family-functioning dimensions were lower than those of the control group at a statistically significant level (*p* < 0.05).

**Figure 1:**
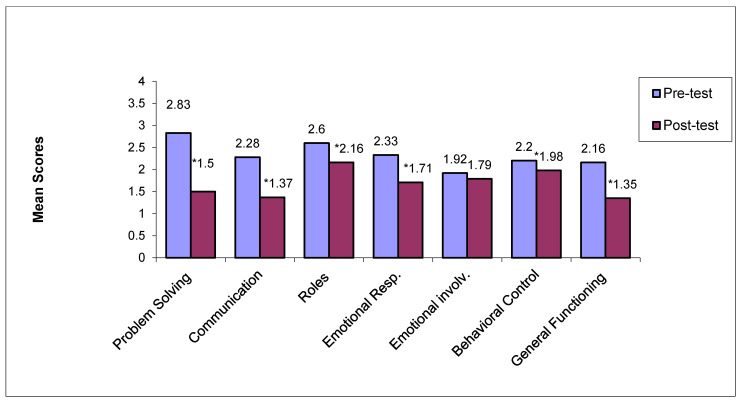
Pre- and post-treatment mean scores across all FAD dimensions within the treatment group. * Statistically significant differences between pre- and post-treatment mean scores for the treatment group (*p* < 0.05).

**Figure 2:**
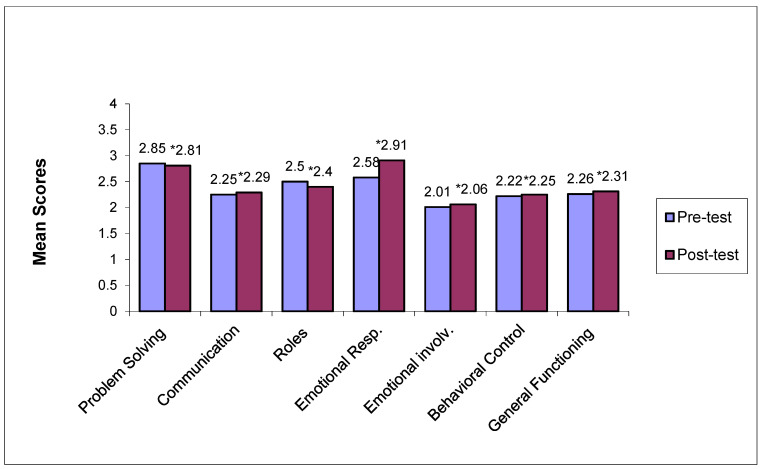
Pre- and post-treatment mean scores across all FAD dimensions within the control group. * Non statistically significant differences between pre- and post-treatment mean scores for the treatment group (*p* > 0.05).

Figure 1 and Figure 2 depict pre- and post-treatment mean scores across all dimensions of family functioning of the FAD for the treatment and the control group, accordingly. Mean score differences on all FAD dimensions for the treatment group before and after treatment were at a statistically significant level (*p* < 0.05), with the exception of emotional involvement (*p* = 0.611). No statistically significant differences were noted for the control group pre- and post-treatment (*p* > 0.05).

#### 3.1.2. Family Rituals Scale (FRS) and Family Burden Scale (FBS)

Table 4 depicts the pre- and post-treatment mean scores (SD) on the FRS and the FBS, as well as on all their subscales, within the treatment group (N = 6) and within the control group (N = 6), and it also depicts the cutoff scores on both scales. In addition, the results of the Wilcoxon matched-pairs signed-rank test analysis (z-values and *p*-values) are depicted in Table 4 for the FRS and FBS total scale and subscales mean score differences within and between groups. The results showed that there was a statistically significant improvement at *p* < 0.05 for the FRS mean scores after treatment for the treatment group, (pre-test M = 20.17; SD = 2.56), post-test M = 17.33; SD = 2.16, z = −2.27, *p* = 0.02), while the control group pre- and post-test mean score differences were of no statistical significance (pre-test M = 22.50; SD = 4.2, post-test M = 23.63; SD = 4.16, z = −1.61, *p* = 0.08). The findings were similar for FBS. Specifically, for the treatment group, there was a statistically significant improvement at *p* < 0.05 for the FBS total mean score after treatment (pre-test M = 21.50; SD= 5.11, post-test M = 13.5; SD = 4.03, z = −2.22, *p* = 0.026), while for the control group, pre- and post-treatment total FBS mean score differences were not statistically significant (pre-test M = 22.30; SD = 3.2, post-test M = 25.55; SD = 3.6, z = −1.02, *p* = 0.07). Subscale pre- and post-test mean score comparisons for the treatment group showed a statistically significant decrease in family burden across three out of four of the family burden dimensions: Social Life (pre-test M = 8.70; SD = 2.7, post-test M = 6.63; SD = 3.9, z = −2.73, *p* = 0.03, Aggressiveness subscale mean scores (pre-test M = 3.30; SD = 2.7, post-test M = 2.37; SD = 1.8, z = −2.6, *p* = 0.04), Health subscale mean scores (pre-test M = 7.25; SD = 2.1, post-test M = 3.13; SD = 2.05, z = −2.17, *p* = 0.011). Nevertheless, the Financial Burden subscale mean scores difference was not statistically significant for the treatment group (*p* > 0.05). For the Control group pre- and post-test, the FBS mean subscale score differences were not statistically significant for any of the four dimensions (*p* > 0.05).

**Figure 3:**
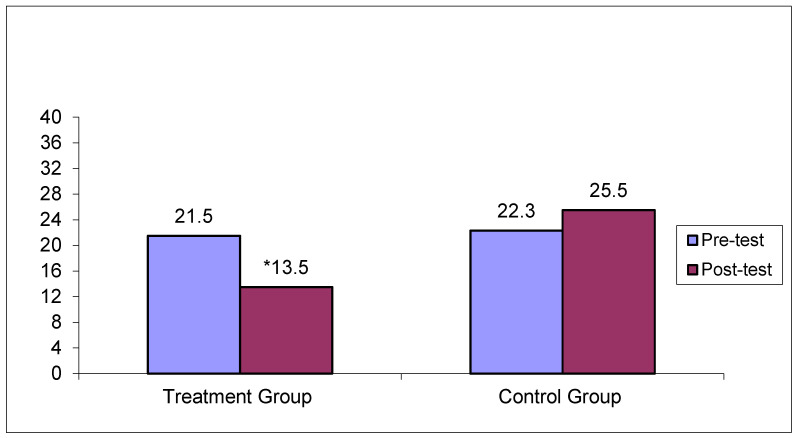
Pre- and post-test total mean scores on the FBS across the treatment and control groups. * Statistically significant differences were only seen between pre- and post-test mean scores for the treatment group (*p* < 0.05).

**Figure 4:**
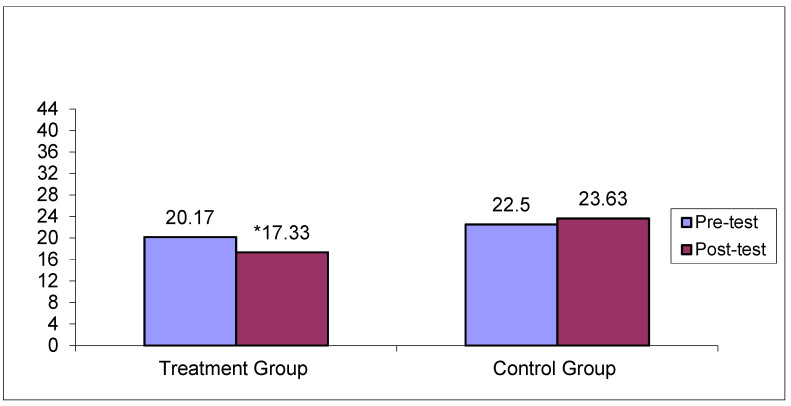
Pre- and post-test total mean scores on the FRS across the treatment and control groups. * Statistically significant differences between pre- and post-test mean scores only for the treatment group (*p* < 0.05).

Table 4 shows the results of the Mann–Whitney U-test comparisons of the total and subscale mean scores on the FRS and the FBS at pre- and post-test, between the treatment group (N = 6) and the control group (N = 6). At the pre-test, there were no statistically significant differences between the two groups (*p* > 0.05), showing no systematic differences in the level of disruption of family rituals and family burden between the two groups. Nevertheless, after the psychoeducational therapeutic intervention, the mean scores between the two groups differed significantly (*p* < 0.05) in favor of the treatment group in both FRS and FBS and its subscales, except for the mean scores on the Financial Burden subscale (*p* > 0.05). Figure 3 and Figure 4 depict pre- and post-treatment total mean scores for the FBS and the FRS scales for the treatment and the control group, respectively.

As shown in Figure 3, the total mean score differences on the FBS scale for the treatment group before and after treatment were at a statistically significant level (*p* < 0.05). No statistically significant mean score differences were noted for the control group pre- and post-treatment (*p* > 0.05). As shown in Figure 4, the total mean score differences on the FRS scale for the treatment group before and after treatment were at a statistically significant level (*p* < 0.05). No statistically significant differences were noted for the control group pre- and post-treatment (*p* > 0.05).

As mentioned in the data collection and analysis section, data were systematically collected on the communication and problem-solving skills of the parents who participated in the treatment group. All parents demonstrated great improvement in all those skills. Interobserver agreement on data collected during parent training ranged from 90 to 100% agreement.

### 3.2. Qualitative Analysis of Parents’ Self-Reports

Table 5 depicts the qualitative analysis conducted on the answers made by parents of the treatment group, which were provided during the semi-structured interviews before and after treatment in relation to knowledge about ASD and stigma management.

#### 3.2.1. Knowledge About ASD

##### Understanding of the Causes of ASD

Parents’ answers regarding the possible causes of ASD, prior to intervention, from both the treatment and the control group (N = 12) may be categorized around two themes: (a) environmental and psychological factors (e.g., “I was working a lot during pregnancy”, “I was spending too much time on the internet”, “My son regressed when he was vaccinated”, “stressful events during pregnancy, like my father’s death”, “I think he took after his father’s personality”, etc.) and (b) vagueness and general confusion (e.g., “It is a very confusing disorder and I find it hard to understand”). After study completion, the parents’ answers in the treatment group (N = 6) shifted toward genetic/neurobiological explanations for the etiology of ASD (e.g., “genetic disorder of a very complex nature”, “It is a brain dysfunction that happened before birth”). Contrary to the treatment group, no thematic changes were detected in the answers of the control group after treatment.

##### Symptomatology of ASD

Before treatment, the parents’ descriptions of their offspring’s symptoms, both in the treatment and the control group (N = 12), were organized around personality characteristics: (e.g., “my child is an introvert”, “He is very stubborn”, etc.). After the intervention, parents in the treatment group (N = 6) were able to describe the main neurodevelopmental characteristics of ASD (difficulties in communication, emotional expression, play and social skills, behavioral issues, etc.), while there were no thematic shifts in the control group.

##### Treatment of ASD

Prior to intervention, parents in both the treatment and the control groups (N = 12) considered medication as the only possible effective treatment, while they were simultaneously seeking a miraculous solution. Following treatment, parents in the treatment group identified the importance of intensive psychoeducational programs for the child and the family (e.g., structure, routines, intensive educational programs, alternative communication programs, and behavior support programs, together with a supportive family atmosphere). No thematic shifts were identified for the control group.

#### 3.2.2. Stigma Management

##### Social Stigma Management

Before the intervention, parents’ answers regarding social stigma management, in both the treatment and the control group, were organized around two main patterns: (a) social withdrawal and the avoidance of public places, and (b) shame regarding their child’s behavior and/or anger at other people staring at or avoiding the child and the family. Following treatment, the parents’ answers in the treatment group (N = 6) shifted toward three major themes: (a) social networking with other families with a child with ASD, (b) strengthening of family cohesion through family outdoor activities, and (c) social networking with relatives and members of their community and a need to inform people about their child’s disability. No thematic changes were detected in the answers of the control group following the introduction of treatment.

##### Self-Stigma Management

Before the intervention, parents’ answers regarding self-stigma management, both in the treatment group and the control group (N = 12), revolved around two main themes: (a) self-blame, guilt, and a sense of failure in the parental role, and (b) increased levels of parental stress due to lack of skills for managing their child’s behavior, meeting financial needs, and planning for their child’s future. After the intervention, parents’ self-reports for the treatment group (N = 6) shifted toward two new themes: (a) a sense of empowerment in the parental role and a need to advocate for their child with a sense of pride for being a parent of a child with ASD and (b) a sense of efficacy in the parental role through a better understanding of the nature of ASD, in general, and their child’s needs, in particular. No thematic changes were identified in the control group.

## 4. Discussion

Families of children with ASD experience unique stressors in their daily lives since autism has pervasive effects across all the domains of child development. The complexity of the disorder, the disruption of family functioning, the social isolation due to social and self-stigmatization, and a host of other factors lead to high stress levels and to family burden [62].

The aim of the present study was to investigate the effectiveness of a long-term, multi-family, group psychoeducational intervention to assist the parents of children with ASD in overcoming the parental and family difficulties associated with the diagnosis of ASD. Specifically, both quantitative and qualitative means of assessment were utilized to assess the effectiveness of the intervention.

The quantitative analysis included three standardized scales that were administered to both the treatment and control groups prior to and after applying the intervention. The results revealed that there were no systematic differences between the two groups prior to intervention. Thus, we may ascertain that certain differences between the two groups, following the intervention, may be attributed to the psychoeducational program that was applied [63].

Following the intervention, systematic changes were not noted in the control group for any of the three standardized scales that were administered. In contrast, several systematic improvements were achieved by the treatment group. Those improvements were statistically significant and may be summarized as follows.

A. On the FAD scale, prior to the intervention, the scores obtained placed family functioning at pathological levels in six out of seven family function subscales for both groups. These results are consistent with the findings of previous studies that report high stress levels, negative emotional intensity, and marital communication and problem-solving difficulties in the parents of children with ASD [6,10,17,18,19,28,33]. Following the intervention, scores within a normal range were obtained on the six aforementioned subscales, but only for the treatment group. Specifically, the following areas were improved: emotional responsiveness, communication, behavior control and allocating roles and responsibilities, and problem-solving. To our knowledge, this is the first study that demonstrates improved communication and problem-solving skills in the parents of children with ASD, following the application of a psychoeducational treatment program, in contrast to prior findings [6,63,64]. As pointed out earlier, a lack of improvement in these two domains may have been attributable to the short duration of the psychoeducational intervention applied in earlier studies. The effectiveness of the present psychoeducational model may be attributed to its duration (long-term application) and to the fact that it included group psychological counseling and social support among group members [64,65,66].

B. On the FRS, prior to the intervention, the scores obtained indicated serious disruption to family rituals and routines. These findings were anticipated since the FRS assesses family engagement in family traditions or religious holidays and the scheduling of trips and family celebrations, as well as the establishment of patterned routines (e.g., eating together on Sundays, cooking special meals, and going out on weekends)—areas in which most families with a child with ASD encounter great disruption [50]. Following the intervention, statistically significant improvements were noted in all the aforementioned areas. These findings are consistent with prior findings pertaining to psychoeducational therapeutic programs applied to families of other clinical populations [25,30].

C. On the *FBS*, which assesses subjective and objective burden, it is worth noting that prior to treatment, parental burden was within a marginally normal range (slightly below the cutoff point). This finding was unexpected, in light of the relevant literature worldwide that underlines the high levels of family burden bestowed due to the strain associated with raising a child with ASD [10,14,67,68,69]. This finding may be attributed to the fact that the children of all families who participated had been receiving behavior-analytic treatment for several years. Thus, the service needs of the children of those families were met to a satisfactory degree, which, according to empirical findings, is an important factor in reducing family burden [70]. Another tentative explanation relates to culturally bound differences, since empirical findings suggest that Mediterranean parents, and especially Mediterranean mothers, refuse to perceive or to admit that they perceive their offspring with a handicap as a “burden” [24,30]. Following the intervention, statistically significant reductions were noted by all parents in terms of (a) family social isolation, (b) behavior outbursts by the child with ASD, and (c) the emergence of psychosomatic health issues as a result of extending the provision of care. This is a crucial finding since there is limited evidence about the effectiveness of group psychoeducation programs in decreasing the objective and subjective burden of families with a member with ASD [33,68,71].

Qualitative data were collected for both the treatment and the control groups and were obtained through individual semi-structured interviews before and after the intervention. Prior to the intervention, no systematic differences were noted between the two groups. Following the intervention, no systematic changes were reported by the control group, whereas several improvements were reported by the participants of the treatment group. Specifically, the following improvements were reported:

A. Parents reported more accurate information about the etiology and the characteristics of ASD and appreciated the importance of early intervention and of parent training in behavior management and problem-solving, with the aim of achieving optimal outcomes. These findings are consistent with the existing literature related to the benefits of psychoeducation and parent training on parental skills and knowledge pertaining to ASD [33,72,73].

B. Thematic analysis of parental reports reflected major improvements in social and self-stigma management. Namely, the parents shifted from parental social withdrawal, avoidance of public places, shame, and embarrassment at their child’s behavior to active social networking with other group members and relatives and a proactive tendency to inform other people about their offspring’s disability, mainly by organizing outdoor activities and by participating actively in public events. Pertaining to self-stigma, parents shifted from self-blame, a sense of failure in the parental role, and fear of social judgment and social rejection to a sense of efficacy in the parental role and a sense of pride for being the parent of a child with ASD. These shifts may work as a buffer against cultural reactions to aberrant behavior (e.g., staring, rude comments, or avoiding interaction) since having a more accurate understanding of ASD is identified as one of the critical factors for empowering families against stigma [74,75].

### Study Limitations and Implications for Future Research

The current study has several limitations. The first limitation pertains to the small size and the non-random selection of the sample. Both of those limitations compromise the generalization or the external validity of the findings. It would be important for future research to replicate the present study with a larger sample.

Another factor that limits the external validity of the present study is the familiarity of the participants with the research settings. Parents’ attendance and commitment to the psychoeducational group sessions were high, which may be attributed to this familiarity and may have led to the establishment of a strong therapeutic alliance between the therapists who led the sessions and the group members. Thus, the findings of the present study may not be generalized to services offered in settings that are unfamiliar to the participating parents. Furthermore, the extent to which the pre-existing therapeutic alliance and familiarity interplayed with the effectiveness of the group intervention is not systematically assessed in this study and remains to be examined in future research [33,50].

Post-treatment assessment was conducted one month following the completion of the intervention, but long-term maintenance was not systematically assessed. There are only anecdotal data that provide support regarding maintenance. Specifically, the participating parents reported that they continued to have a closer and mutually supportive relationship with their spouses. In addition, the participating couples reported that they developed social ties among themselves. It would be important to investigate whether long-term psychoeducation could possibly help parents to maintain self-determination, a sense of coherence, family empowerment, peer-to-peer support, and long-lasting coping with stigma.

The duration of the group therapeutic intervention was over 12 months and was carried out on a bi-weekly basis. It would be important to investigate whether a more cost-effective, short-term group psychoeducational program could lead to similar outcomes. According to prior research, it was suggested that a minimum of an 18-month duration was necessary for the therapeutic effects of a psychoeducational program to be maintained [38]. In addition, manualizing the treatment protocol may contribute to the replication of the present study with greater precision [33]. In summary, future research efforts may address issues that improve the external validity of the findings and assess the effectiveness of the different parameters of the intervention as a means of building group family psychoeducational interventions that address the needs of families including children with ASD. Finally, it would be worth investigating sociodemographic and other family or child variables (e.g., age or severity of difficulties) that may contribute to treatment outcomes.

## 5. Conclusions

This study aimed to fill a research gap in supporting the families of children with ASD through the application of a group psychoeducation intervention that was designed to address specifically the needs of such families and that draws upon prior evidence-based research with families including members with other chronic disorders. The analysis of both the quantitative and qualitative data of this pilot study replicates prior findings about the importance of psychoeducational interventions and provides evidence that long-term group family psychoeducation, promoting a better awareness of ASD and its treatment, the development of effective communication and problem-solving skills among family members, and the management of stigma may greatly improve family dynamics in terms of improving engagement in family rituals and routines and overall family functioning, as well as minimizing family burden, which are all factors that are positively associated with the family’s quality of life and may serve as the foundation for social rehabilitation of families that raise a child with ASD.

## Figures and Tables

**Table 1:** Sociodemographic characteristics of the participating parents and their offspring.

	Treatment Group N = 6		Control Group N = 6		Total Sample N = 12	
Sociodemographic Characteristics of Parents	M, or N	SD, or %	M, or N	SD, or %	M, or N	SD, or %
Fathers	3	50%	3	50%	6	50%
Mothers	3	50%	3	50%	6	50%
Age (M ± SD)	40.83 ± 3.66		41.17 ± 4.6		41.00 ± 3.9	
Years of formal education (M ± SD)	14.67 ± 4.0		13.33 ± 2.8		14.00 ± 3.3	
**Sociodemographic** **Characteristics** **of Offspring with ASD**						
Age (M ± SD)	7.34 ± 2.55		6.45 ± 3.4		7.11 ± 3.2	
Years since the initial diagnosis (M ± SD)	5.00 ± 0.8		4.33 ± 1.3		4.67 ± 1.1	
Received specialized treatment services	ISBA			DCH II		
Intensity of treatment	<3 h per day			<3 h per day		

**Table 2:** Content and structure of group psychoeducational intervention (treatment group).

Topics per Session	Sessions
Pre-test assessment	
Semi-structured interviews with each member of the two groups to assess: Basic knowledge about ASD prior to intervention Family needs and family social support systems Individual and family goals of each interviewee Administration of the following scales: Family Assessment Device, Family Rituals Scale, and Family Burden Scale Open-ended, semi-structured interview about the nature, causes, and treatment of ASD, and social and self-stigma management	1 session per participant
**Treatment group psychoeducational therapeutic program**	**Duration** **(in 90-min sessions)**
Introduction—engagement: Introduction of the group members and establishment of the therapeutic alliance, with the negotiation of common goals, roles, and responsibilities. Participants signed a therapeutic contract.	1 group session
2. Focus group on the nature, risk factors, and available interventions of ASD (focus group).	1 group session
3. Information on the nature, causes, and treatment of ASD (provision of handouts).	3 group sessions
4. Self- and social stigma management: Discussion of psychosocial aspects of ASD among group members (focus group).	3 group sessions
5. Communication skills training using modeling, role-playing, and positive or corrective feedback: How to express positive emotions Active listening How to express negative emotions How to ask politely (Handouts with homework assignments).	10 group sessions
6. Problem-solving skills training: Crisis management Positive behavior support techniques	5 group sessions
7. One-month follow-up session on the maintenance of communication and problem-solving skills.	1 group session
**Total number of treatment sessions**	**23 group sessions**
**Post-test assessment**	
Semi-structured interviews with each member of the two groups in order to assess: Basic understanding of ASD after the intervention Review of family needs and family-support systems Review of Individual and family goals of the interviewee Administration and completion of FAD, FRS, and FBS questionnaires Administration of an open-ended semi-structured questionnaire related to the nature, causes, and treatment of ASD, and of social and self-stigma management.	1 session per participant

**Table 3:** Mean scores on the FAD subscales pre and post-intervention for the treatment group (N = 6) and the control group (N = 6).

	Treatment Group (N = 6)			Control Group (N = 6)			Average Rank Between the Two Groups Comparisons **			
FAD Subscales	Pre-Test Mean (±SD)	Post-Test Mean (±SD)	z Value * (*p* < 0.05)	Pre-Test Mean (±SD)	Post-Test Mean (±SD)	z Value * (*p* < 0.05)	Treatment Group (N = 6)		Control Group (N = 6)	*p*
Problem-solving Cut-off = 2.20	2.83 (0.37)	1.50 (0.18)	−2.22 (*p* = 0.011)	2.85 (0.4)	2.81 (0.23)	–1.83 (*p* = 0.08)	Pre	8.39	8.61	0.668
							Post	5.50	12.50	0.001
Communication Cut-off = 2.20	2.28 (0.52)	1.37 (0.29)	–2.21 (*p* = 0.017)	2.25 (0.5)	2.29 (0.3)	–0.61 (*p* = 0.32)	Pre	7.21	7.65	0.773
							Post	3.50	11.20	0.01
Roles Cut-off = 2.30	2.6 (0.3)	2.16 (0.3)	–2.20 (*p* =0.011)	2.5 (0.3)	2.4 (0.3)	–0.41 (*p* = 0.43)	Pre	9.56	7.44	0.342
							Post	8.42	8.58	0.08
Emotional response Cut-off = 2.20	2.33 (0.7)	1.71 (0.5)	–1.68 (*p* = 0.011)	2.58 (0.3)	2.91 (0.4)	–1.73 (*p* = 0.16)	Pre	8.44	8.56	0.923
							Post	4.81	8.19	0.001
Emotional involvement Cut-off = 2.10	**1.92 (0.2)**	1.79 (0.1)	–2.03 (*p* = 0.611)	2.01 (0.3)	2.06 (0.4)	–1.41 (*p* = 0.72)	Pre	9.63	9.38	0.382
							Post	5.00	9.12	0.002
Behavioral control Cut-off = 1.90	2.20 (0.2)	*1.98 (0.1)*	–1.92 ((*p* = 0.04)	2.22 (0.2)	2.25 (0.2)	–0.32 (*p* = 0.12)	Pre	8.38	8.14	0.959
							Post	6.15	8.08	0.05
General Functioning Cut-off = 2.00	2.16 (0.3)	1.35 (0.2)	–2.03 (*p* =0.012)	2.26 (0.3)	2.31 (0.3)	–0.08 (*p* = 0.33)	Pre	8.18	8.13	0.738
							Post	5.44	8.56	0.007

* Wilcoxon matched-pair signed-rank test comparisons of pre- and post-test scores within the treatment group and within the control group. Statistical significance was set at *p* < 0.05; ** Mann–Whitney U-test comparisons between the two groups at pre-test and post-test. Statistical significance set at *p* < 0.05.

**Table 4:** Mean scores on the FRS and FBS and its subscales pre- and post-intervention for the treatment group (N = 6) and the control group (N = 6).

Scales	Treatment Group (N = 6)			Control Group (N = 6)			Average Rank Between the Two Groups’ Comparisons **			
	Pre-Test Mean (±SD)	Post-Test Mean (±SD)	z Value * (*p* < 0.05)	Pre-Test Mean (±SD)	Post-Test Mean (±SD)	z Value * (*p* < 0.05)	Treatment Group (N = 6)		Control Group (N = 6)	*p*
**FRS Total** Cutoff score = 18	20.17 (2.56)	17.33 (2.16)	–2.27 (*p* = 0.027)	22.50 (4.2)	23.63 (4.2)	–1.61 (*p* = 0.08)	Pre	6.89	6.43	0.77
							Post	4.38	12.23	0.01
**FBS Total** Cutoff score = 24	21.50 (5.11)	13.5 (4.03)	–2.22 (*p* = 0.026)	22.30 (3.2)	25.55 (3.6)	–1.02 (*p* = 0.06)	Pre	7.23	8.09	0.65
							Post	6.62	11.94	0.02
FBS Social life	8.7 0 (2.7)	6.63 (3.9)	–2.73 (*p* = 0.03)	9.53 (2.3)	10.20 (2.2)	–0.33 (*p* = 0.14)	Pre	7.01	7.19	0.89
							Post	6.19	10.31	0.04
FBS Aggressiveness	3.30 (2.7)	2.37 (1.8)	–2.6 (*p* = 0.04)	3.47 (4.2)	3.80 (2.6)	–0.15 (*p* = 0.52)	Pre	6.54	6.76	0.89
							Post	5.10	11.00	0.01
FBS Health	7.25 (2.1)	3.13 (2.5)	–2.7 (*p* = 0.011)	7.63 (3.5)	8.00 (2.6)	–0.82 (*p* = 0.14)	Pre	8.55	8.14	0.83
							Post	5.04	10.54	0.01
FBS Financial	2.25 (1.5)	1.87 (1.3)	–1.4 *(p > 0.05)*	2.37 (1.3)	2.55 (1.3)	–0.34 (*p* = 0.53)	Pre	7.79	8.01	0.92
							Post	7.12	8.31	0.69

* Wilcoxon matched-pair signed-rank test comparisons of pre- and post-test scores within the treatment group and within the control group. Statistical significance is set at *p* < 0.05; ** Mann–Whitney U-test comparisons between the two groups at pre-test and post-test. Statistical significance is set at *p* < 0.05.

**Table 5:** Pre- and post-treatment results: parents’ self-reports related to comprehension of the nature, causes, and treatment of ASD and social and self-stigma management.

1. Knowledge About ASD				
	Before N = 12 (Common Themes for Both Treatment and Control Group)		After N = 6 (Only Treatment Group/No Pattern Shift for Control Group)	
Areas	Themes	Example Quotes	Themes	Example Quotes
**1.1. Causes**	-Psychological -Environmental	“I was stressed out during pregnancy, because of my father’s death” “I spent too much time on the internet” “I was working long hours”	-Neurobiological -Genetic nature	“Genetic disorder of a very complex nature” “It is a brain dysfunction that happened before birth” “It is a metabolic disorder—an infection of the brain”
	-Confusion -Luck or destiny	“For me it is a confusing disorder that I find hard to understand” “Nobody knows, it was meant to happen to us”		
**1.2. Symptoms**	Personality traits	“My child is an introvert person” “He is very self-absorbed” “He is very immature” “He is very stubborn” “He does not take no for an answer”	Neurodevelopmental characteristics	“It is a developmental disorder that affects behavior at many levels (communication, emotional expression, play skills, social relations, self-help skills” “It is a neurological health issue. My daughter cannot communicate what she wants, and this is why she has a lot of behavior issues”
**1.3. Treatment**	-Medical solution -Miracle	“I hope for a miracle cure” “I pray to God, every day, for him to get well”	-Psychoeducational programs for the child and the family	“I believe in intensive structured educational programs” “I believe in structure and everyday routines in conjunction with a supportive family atmosphere”
**2. Stigma Management**				
**2.1. Social stigma**	-Social withdrawal -Shame, anger, guilt	“I avoid going to the playground with my child” “We are not invited anymore by relatives during the holidays” “I often feel embarrassed when I am in public places with my child “I feel that other people feel sorry for me” “I get really angry when people are staring at us”	-Social networking within the group -Family activities -Social networking with the community and relatives -Need to educate the community about ASD	“I really enjoyed spending the holidays with one of the other families that I met during the group program” “We are planning a family summer vacation” “We have invited my brother’s family over for Christmas” “I now believe that people understand how difficult raising a child with ASD might be and that they respect me” “I believe that ignorance is the reason for social stigma and that we should inform people about our child’s ASD”
**2.2. Self-stigma**	-Sense of failure as a parent -Self-blame, self-pity -Increased parental stress	“I believe that god is punishing me” “I constantly feel guilty for not doing enough for my child” “I feel that everything is lost” “I feel stressed, wondering whether there is anything else I can do for my child that I cannot financially afford” “I really don’t know how to handle his behaviors” “I am really worried about the future”	-Empowerment -Need for advocacy	“I am very proud that I have a special child, and I think that my son is proud of his parents too” “I really don’t care how other people see us. I just want my child to be happy”
			-Satisfaction from the parental role	“I feel that we make one small step forward, every day” “As a father I feel that I respond more and more to my child’s needs”

## Data Availability

The raw data supporting the conclusions of this article are unavailable due to privacy and the ethical restrictions applied because of the sensitivity of parental reports and the small size of the research sample.

## Abbreviations

The following abbreviations are used in this manuscript:

ASD	Autism spectrum disorder
ISBA	Institute of Systemic Behavior Analysis
DCH II	Day Center Hara II
FAD	Family Assessment Device
FRS	Family Ritual Scale
FBS	Family Burden Scale
NKUA	National and Kapodistrian University of Athens
